# Stimulants

**Published:** 1874-11

**Authors:** 


### STIMULANTS.

All nations, in all ages, and from the earliest times, have had stimulants peculiar to themselves; these stimulants are artificial, indicating that the man under all circumstances and surroundings has been driven by some strong power to devise some drink which shall elevate both body and mind above certain depressed conditions. The poor and the ignorant of all countries fall under the influence of liquor in much greater numbers than the better classes, because the causes of depression and discouragement are more numerous and more abiding, while they have not the mental resources of the better educated, nor the means of diversion which the well-to-do have at their command; and for these reasons the drunken poor, however degraded, ought to have the sympathy and the aid of every humane friend.

There are times when troubles of one kind or another are so heavy, that a man feels as if he would die; some do, others commit suicide, while the many resort to drink as a means of forgetfulness and of elevation of spirits, even if it be temporary. Persons under the lash, or the unendurable tortures of the inquisition, have prayed with desperate energy, and have received with inexpressible gratitude a single moment's cessation of the fearful inflictions, knowing, at the same time, that it is only protracting their agony, without diminishing the number of the stripes or the turns of the wheel; and so those who drink, while they know that the same trouble will be present after the influence of the drink passes away, choose the temporary oblivion.

At the same time, there is a method of obtaining a forgetfulness of sorrow, of diminishing the weight of trouble, and of alleviating mental distress, from whatever cause, a thousand times preferable to vulgar liquor drinking, and that is, engaging in something so pressing in its nature, or so widely different in its mental influences, as to compel the mind away to other thoughts. In that frame, if you are in trouble, bury yourself in something else, and do not mope and fret or abandon yourself to moodiness in the retiracy of your own chamber, in the darkness of the night or in the seclusion of the forest. If nothing else offers, take a rapid and determined walk down the Broadway, or Chestnut street, or the Fifth Avenue of your village; this is a prescription which costs no money, and will never fail of favorable results.

The habit of taking stimulants for mental depression or bodily plague always tends towards confirmed drunkenness, because nature, being once helped, increases her demands with the remorselessness of inexorable fate. Hence the man who begins with lager beer, soon finds himself craving wine and whisky, ending in Brandy, Arack, or Absynthe. The following table shows the different amounts of Alcohol, that is, the power of inducing drunkenness, in the various beverages of the times, enabling a man to determine how fast he is going to destruction, and how near he is to the verge of the horrible pit. When it is said that whisky has fifty per cent. of Alcohol, it means that it is half water, &c. The proportions are given in round numbers, as being more readily remembered:

| | | |
|---|---|---|
| **BEER.** | | Contains of Alcohol. |
| Schenck's Beer, - - | 2 | per cent. |
| Ale, - - - | 3 | " |
| Lager Beer, - - | 4 | " |
| Brown Stout, - - | 5 | " |
| London Porter, - - | 5 | " |
| Scotch Ale, - - | 9 | " |
| **WINES.** | | |
| Apple new Wine or Cider, | 5 | " |
| Claret Wine, - - | 7 | " |
| Rudesheimer, - - | 7 | " |
| Madeira, - - | 14 | " |
| Malaga, - - | 15 | " |
| Port, - - | 15 | " |
| Currant, - - | 15 | " |
| **BITTERS.** | | |
| Pierce's Bitters, - - | 6 | " |
| Vinegar Bitters, - - | 8 | " |
| Wheeler's Wine Bitters, | 15 | " |
| Temperance Bitters, - | 17 | " |
| Oxygenated Bitters, - | 18 | " |
| California Bitters, - | 19 | " |
| Hoffman's Bitters, - | 21 | " |
| Restorative Bitters, - | 21 | " |
| Clark's Wine Bitters, - | 22 | " |
| Peruvian Bitters, - | 22 | " |
| Quaker Bitters, - | 23 | " |
| Langley's Bitters, - | 24 | " |
| Speers' Bitters, - | 25 | " |
| Baker's Bitters, - | 26 | " |
| Atwood's Bitters, - | 26 | " |
| Puritan Bitters, - | 26 | " |
| Webber's Bitters, - | 27 | " |
| Hartshorn Bitters, - | 27 | " |
| Warren's Bitters, - | 30 | " |

| | | Contains of Alcohol. | |
|---|---|---|---|
| Colton's Bitters, | - | 30 | per cent. |
| Davis' Bitters, | - | 30 | " |
| Plantation (Drake's) Bitters | | 30 | " |
| Sweet's Bitters, | - | 31 | " |
| Fisch's Bitters, | - | 32 | " |
| Arrington's Bitters, | - | 33 | " |
| Golden Seal Bitters, | - | 34 | " |
| Rush's Bitters, | - | 34 | " |
| Wild Cherry Bitters, | - | 35 | " |
| Mishler's Bitters, | - | 36 | " |
| Franks' Panacæ Bitters, | - | 37 | " |
| Atwood's Tonic Bitters | - | 40 | " |
| Boker's Bitters, | - | 41 | " |
| Hostetter's Bitters, | - | 43 | " |
| Richardson's Bitters, | - | 59 | " |

Thus it is seen that while persons are using Bitters as a medicine, and speak of taking "nothing but Bitters," they are often drinking three times a day a more concentrated form of Alcohol than is found in the purest Whiskies and Brandies, and in so doing find themselves confirmed hard drinkers before they are aware of it. It should be set down as a settled rule that "Bitters" in any form is disguised Whisky.

| | | | Contains of Alcohol. | |
|---|---|---|---|---|
| Gin, | - | - | 30 | per cent. |
| Rum, | - | - | 36 | " |
| Brandy, | - | - | 38 | " |
| Whisky, | - | - | 40 | " |
| Proof Spirit, | - | - | 49 | " |
| Alcohol, | - | - | 92 | " |

Taking a pint for a pound, three bottles of Claret equal a pint of Whisky. A Frenchman easily consumes a bottle of Claret at dinner; hence we may talk as much as we please about wine-drinking countries not having many drunken people, yet here is a plain fact, that many take at dinner as much Alcohol as is found in a "tumbler-full" of Whisky, and the man who does that must be near his ruin.

AN INSIDIOUS MISTAKE.

Some persons congratulate themselves in having given up Whisky drinking, when they have only substituted for it opium, which has the advantage of being taken with much less liability to observation; but the principle is the same, whether a man is drunk on opium or whisky, and the ill results are as disastrous in the end. A man who can't do without opium, is no better off than one who cannot do without liquor. From the fact that opium can be taken with less risk of notice than liquor, druggists of large cities account for the enormous increase of opium importation within the last few years, and the lamentable announcement is made that a vast majority of opium consumers are women. Let any lady reading this, who is conscious of ever having purchased opium, even once, for personal use, heed the warning that she is in danger of a degradation and a death worse than that of a beastly drunkard.

